# ArCH: improving the performance of clonal hematopoiesis variant calling and interpretation

**DOI:** 10.1093/bioinformatics/btae121

**Published:** 2024-03-14

**Authors:** Irenaeus C C Chan, Alex Panchot, Evelyn Schmidt, Samantha McNulty, Brian J Wiley, Jie Liu, Kimberly Turner, Lea Moukarzel, Wendy S W Wong, Duc Tran, J Scott Beeler, Armel Landry Batchi-Bouyou, Mitchell J Machiela, Danielle M Karyadi, Benjamin J Krajacich, Junhua Zhao, Semyon Kruglyak, Bryan Lajoie, Shawn Levy, Minal Patel, Philip W Kantoff, Christopher E Mason, Daniel C Link, Todd E Druley, Konrad H Stopsack, Kelly L Bolton

**Affiliations:** Department of Medicine, Washington University School of Medicine, St. Louis, MO 63110, United States; Department of Medicine, Washington University School of Medicine, St. Louis, MO 63110, United States; Department of Medicine, Washington University School of Medicine, St. Louis, MO 63110, United States; Invitae Corporation, San Francisco, CA 94103, United States; Department of Medicine, Washington University School of Medicine, St. Louis, MO 63110, United States; Department of Medicine, Washington University School of Medicine, St. Louis, MO 63110, United States; Department of Medicine, Washington University School of Medicine, St. Louis, MO 63110, United States; Department of Medicine, Memorial Sloan Kettering Cancer Center, New York City, NY 10065, United States; Division of Cancer Epidemiology and Genetics, National Cancer Institute, Bethesda, MD 20814, United States; Department of Medicine, Washington University School of Medicine, St. Louis, MO 63110, United States; Department of Medicine, Washington University School of Medicine, St. Louis, MO 63110, United States; Department of Medicine, Washington University School of Medicine, St. Louis, MO 63110, United States; Division of Cancer Epidemiology and Genetics, National Cancer Institute, Bethesda, MD 20814, United States; Division of Cancer Epidemiology and Genetics, National Cancer Institute, Bethesda, MD 20814, United States; Department of Genomic Applications, Element BioSciences, San Diego, CA 92121, United States; Department of Genomic Applications, Element BioSciences, San Diego, CA 92121, United States; Department of Genomic Applications, Element BioSciences, San Diego, CA 92121, United States; Department of Genomic Applications, Element BioSciences, San Diego, CA 92121, United States; Department of Genomic Applications, Element BioSciences, San Diego, CA 92121, United States; Department of Medicine, Memorial Sloan Kettering Cancer Center, New York City, NY 10065, United States; Department of Medicine, Memorial Sloan Kettering Cancer Center, New York City, NY 10065, United States; Department of Physiology and Biophysics, Weill Cornell Medical College, New York City, NY 10065, United States; Department of Medicine, Washington University School of Medicine, St. Louis, MO 63110, United States; Mission Bio, San Francisco, CA 94080, United States; Department of Epidemiology, Harvard T. H. Chan School of Public Health, Boston, MA 02115, United States; Clinical and Translational Epidemiology Unit, Massachusetts General Hospital and Harvard Medical School, Boston, MA 02130, United States; Department of Medicine, Washington University School of Medicine, St. Louis, MO 63110, United States

## Abstract

**Motivation:**

The acquisition of somatic mutations in hematopoietic stem and progenitor stem cells with resultant clonal expansion, termed clonal hematopoiesis (CH), is associated with increased risk of hematologic malignancies and other adverse outcomes. CH is generally present at low allelic fractions, but clonal expansion and acquisition of additional mutations leads to hematologic cancers in a small proportion of individuals. With high depth and high sensitivity sequencing, CH can be detected in most adults and its clonal trajectory mapped over time. However, accurate CH variant calling is challenging due to the difficulty in distinguishing low frequency CH mutations from sequencing artifacts. The lack of well-validated bioinformatic pipelines for CH calling may contribute to lack of reproducibility in studies of CH.

**Results:**

Here, we developed ArCH, an Artifact filtering Clonal Hematopoiesis variant calling pipeline for detecting single nucleotide variants and short insertions/deletions by combining the output of four variant calling tools and filtering based on variant characteristics and sequencing error rate estimation. ArCH is an end-to-end cloud-based pipeline optimized to accept a variety of inputs with customizable parameters adaptable to multiple sequencing technologies, research questions, and datasets. Using deep targeted sequencing data generated from six acute myeloid leukemia patient tumor: normal dilutions, 31 blood samples with orthogonal validation, and 26 blood samples with technical replicates, we show that ArCH improves the sensitivity and positive predictive value of CH variant detection at low allele frequencies compared to standard application of commonly used variant calling approaches.

**Availability and implementation:**

The code for this workflow is available at: https://github.com/kbolton-lab/ArCH.

## 1 Introduction

With normal aging, tissues accumulate acquired somatic mutations (Jacobs *et al.* 2012, Genovese *et al.* 2014, Jaiswal *et al.* 2014, Bolton *et al.* 2020). Mutations in a stem or progenitor cell conferring a fitness advantage can lead to clonal expansion. A common theme of somatic profiling is that the types of mutations leading to a proliferative advantage in normal stem cells often overlap those driving cancer in the respective tissue (Haferlach *et al.* 2014, Jaiswal *et al.* 2014, Martincorena *et al.* 2015, 2018). In some instances, these clonal expansions represent pre-cursor states for cancer. Characterization of the environmental and germline processes that influence clonal evolution of somatic mutations provides a unique opportunity to study evolutionary processes underlying carcinogenesis. The acquisition of somatic mutations in hematopoietic stem and progenitor stem cells (HSPCs) with resultant clonal expansion is called clonal hematopoiesis (CH). CH is generally present at low variant allele fractions (VAF) with the prevalence increasing with each decade of life. CH is associated with a higher risk of hematologic malignancies and to other adverse health outcomes including most notably cardiovascular disease (Jaiswal *et al.* 2014, 2017, Vlasschaert, *et al.* 2023). Longitudinal studies of individuals with CH have elucidated the role of cell intrinsic and extrinsic processes influencing CH progression to hematologic malignancy (Bolton *et al.* 2020, Fabre *et al.* 2022).

Unfortunately, distinguishing CH mutations from artifacts remains a challenging problem. There is a lack of validated standards for variant calling and filtering and differences in CH calling can lead to divergent conclusions regarding CH associations (Bick *et al.* 2020a, Vlasschaert, *et al.* 2023). These challenges are even more amplified for low VAF mutations which have resulted in most CH studies to date, primarily focused on the detection of mutations above 2% VAF. However, the use of advanced error correction in high depth sequencing have made the detection of low VAF CH mutations possible (Kivioja *et al.* 2011, Young *et al.* 2016). In fact, several studies have already shown that most CH clones are present at low VAFs (<2%) (Young *et al.* 2015, 2019, Friedman *et al.* 2023) and indeed are ubiquitous among adults. Low VAF CH has been shown to increase the risk of leukemia years later (Young *et al.* 2019). The clinical relevance of low VAF acquired mutations in healthy tissues is not limited to the blood; high-depth sequencing studies in the skin (Wei, *et al.* 2021), esophagus (Martincorena, *et al.* 2015, 2018), and liver (Wong, *et al.* 2023) suggest that low VAF clonal mutations contribute to disease. Continued used of high depth sequencing will help us further clarify key unanswered questions, including how the timing of the mutational events and cell extrinsic factors influence clonal selection and progression to cancer. Despite the technical advances in sequencing, variant calling of low VAF CH mutations remains challenging due to a poor signal-to-noise ratio. Robust approaches optimized to CH variant calling, particularly at low VAF, are needed to better identify and study how CH trajectories influence health outcomes.

Here, we describe the development, validation, and performance of an Artifact filtering and Clonal Hematopoiesis calling pipeline, ArCH, which we designed to improve performance, accuracy and sensitivity of CH variant calling including variants at low VAFs (<2%). ArCH is an end-to-end pipeline that leverages UMI-based error corrected consensus sequence building, multiple somatic variant callers and post-variant calling filters to improve the accuracy of variant calling for any targeted sequencing panel. In addition, to facilitate variant interpretation for clinical studies, we developed a functional annotation pipeline for the identification of putative-driver CH variants based on prior literature, expert knowledge databases and somatic cancer mutation databases. Here, we show that ArCH can detect variants at low VAF (0.1%–1%) with high sensitivity and positive predictive value (PPV) using deep targeted sequencing datasets generated from acute myeloid leukemia (AML) tumor: normal dilutions (91.0% sensitivity; 83.0% PPV) and additional independently sequenced data with orthogonal validation (82.0% sensitivity; 95.0% PPV), while retaining 100% sensitivity and PPV for variants at higher VAF (>5%).

## 2 Materials and methods

### 2.1 Samples

To evaluate the accuracy and sensitivity of variant callers to detect low VAF variants, we created a serial dilution series using blood samples from six adults with AML and matched skin samples. Genomic DNA from AML blood and skin samples were extracted using DNeasy Blood and Tissue Kit (Qiagen, Hilden, Germany). Tumor genomic DNA was diluted with skin genomic DNA at a ratio of 1:10, 1:100, 1:1000, and 1:5000. As a second validation set, we analyzed 31 blood samples from 19 adults [12 male participants of the Health Professionals Follow-up Study (HPFS) cohort not selected for any chronic disease, donated in 1993–1995, and 7 samples from adult solid tumor patients]. We refer to this set as the “normal blood” validation set. Genomic DNA was isolated from PBMCs as above. As a third validation set, we included 21 healthy blood samples from HPFS study in which two technical replicates of genomic DNA were isolated from the same sample as described above. As a fourth validation set, we included five blood samples from solid tumor patients in which at least two technical replicates of genomic DNA were isolated from the same sample as described previously. As a set of technical controls (i.e. panel of normal; PoN), we obtained blood samples and extracted genomic DNA as above from seven children (<15 years old) and 20 young adults (<40 years old).

### 2.2 Sequencing

#### 2.2.1 ArcherDX VariantPlex

All samples from the AML tumor:normal dilution series were sequenced using a custom designed DNA sequencing assay (VariantPlex, ArcherDX Inc., Integrated DNA Technologies, Boulder, CO, USA). The panel (23 650 bp) captures the nine most commonly mutated genes in solid tumor patients with CH (Bolton *et al.* 2020) including the full exonic regions of five genes (*DNMT3A, TET2, ASXL1, TP53, CHEK2*) and partial exonic regions of four genes (*JAK2, SRSF2, SF3B1, PPM1D*) (Supplementary Table S1). Libraries were prepared from 250 ng genomic DNA using the VariantPlex protocol, which uses Anchored Multiplex PCR (AMP) technology to generate target-enriched sequencing-ready libraries. Following DNA fragmentation, ligation with a universal ArcherDX molecular barcode adapter was performed, tagging each DNA molecule with a unique molecular index (UMI) and allowing for unidirectional amplification of the sample using gene-specific primers. Libraries were sequenced on a NovaSeq 6000 instrument (Illumina Inc., San Diego, CA, USA), per manufacturer’s instructions. Sequencing was performed to yield a mean coverage of 151 324× prior to consensus building, 26 232× after consensus building and 19 664× following the sequence filtering with 90.3% and 9.7% of the samples achieving at least 80.0% of the targeted regions covered by 10 000× and 20 000× coverage, respectively.

#### 2.2.2 Orthogonal sequencing of normal blood validation set

We re-sequenced the 31 normal blood samples using two different orthogonal approaches. First, samples were sequenced using MyeloSeq™-HD, which uses amplicon capture-based enrichment with UMIs using the HaloPlex Target Enrichment System (Agilent Technologies, Santa Clara, CA, USA). The majority (91.6%) of the target captured by our custom ArcherDX panel is also covered by MyeloSeq™-HD. Libraries were sequenced on a NovaSeq 6000 instrument. Due to the MyeloSeq^TM^-HD panel having a larger region of coverage (313 592 bp), we sequenced to a lower depth, resulting in a mean of 76 148 309 reads per sample and a mean coverage of 2009×.

Second, the same 31 libraries that were generated using MyeloSeq™-HD were re-sequenced with a different sequencing technology—“avidity sequencing” using the Element AVITI™ System (Element Biosciences, San Diego, CA, USA). In brief, for sequence detection, a dye-nucleotide construct called “Avidite,” is made consisting of a dye-labeled core and multiple arms with the nucleotide at the end (Arslan *et al.* 2023). Each of the four Avidite types (A, C, G, T) is labeled with a different dye. The flow cell has the capacity to generate ∼800M-1B polonies (polymerase colonies), with each polony consisting of many copies of the same DNA template. The Avidite arms bind multiple copies within a polony and corresponding fluorescence of nucleotide is detected. Low-binding surface chemistry drives high accuracy while rolling circle amplification eliminates PCR error propagation and limits AT/GC bias. Sequencing was performed to yield a mean of 2396× coverage.

#### 2.2.3 Duplex sequencing

Five blood samples from solid tumor patients were sequenced in replicate using duplex sequencing (TwinStrand Biosciences, Seattle, WA, USA) of 31 common myeloid neoplasm driver genes, which included common CH genes (73 253 bp). Libraries were sequenced on a NovaSeq 6000 instrument with a mean of coverage of 17 044× following consensus sequence building.

### 2.3 ArCH pipeline development

**Figure 1.**
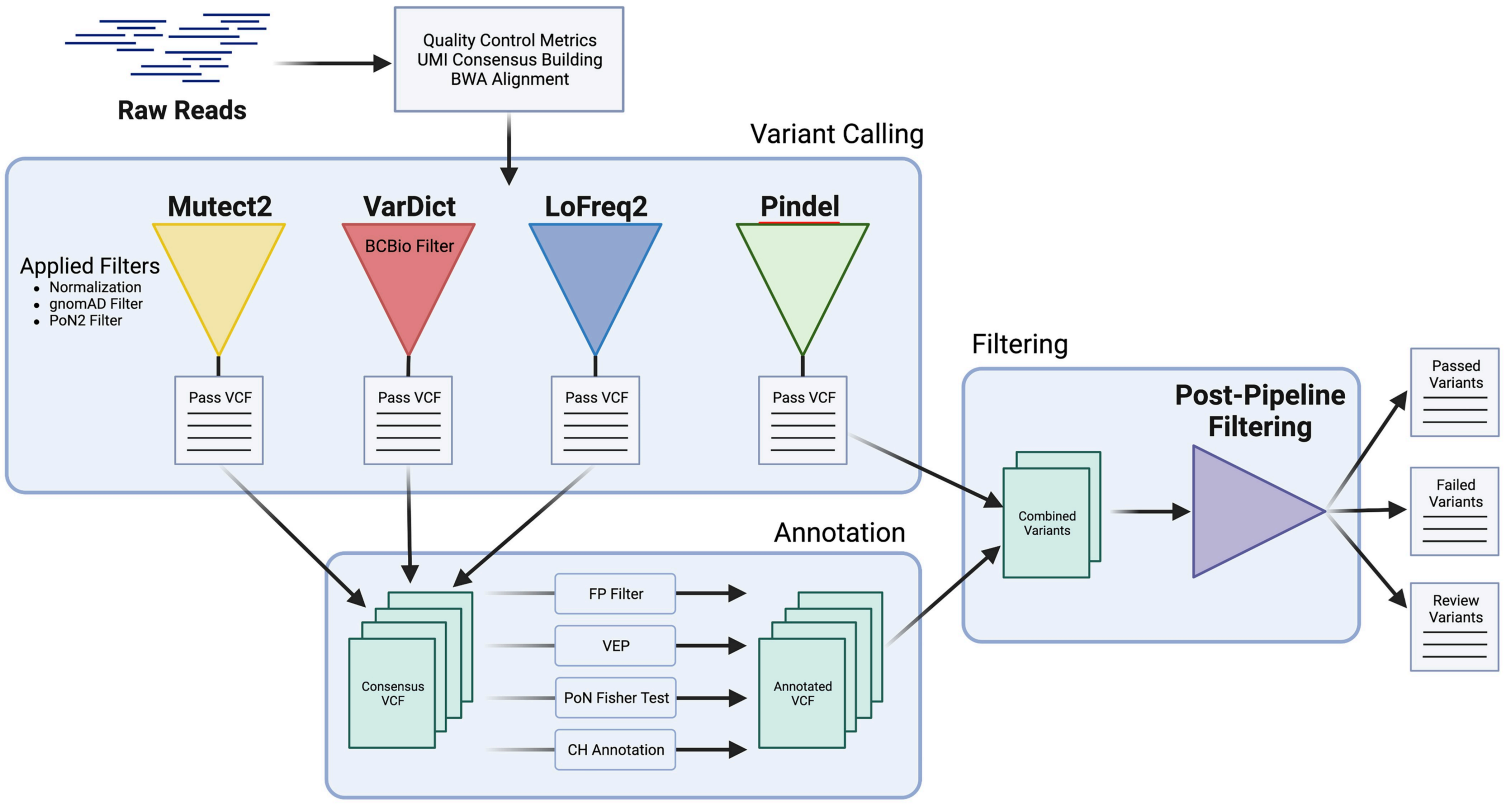
ArCH Workflow. ArCH workflow for multiple variant consensus calling, sequencing artifact filtering, and putative driver annotation. ArCH consists of four general steps: (i) UMI-based error corrected consensus sequence building, (ii) variant calling using multiple variant callers with subsequent normalization and pre-filtering, (iii) annotation of passed variants with pathogenicity and other relevant information, and (iv) false positive filtering and variant sorting into pass, fail, and review. Filtering steps and annotation are performed throughout the workflow to allow for greater optimization and parallelization. Four main annotations are applied onto all passed variants: (i) FP filter—a collection of false positive filters defined in our methods section, (ii) VEP—Variant Effect Predictor that annotates the effect of the variants on genes, transcripts, and protein sequences, (iii) PoN Fisher Test—The statistical calculation of signal-to-noise for each variant relative to our panel of normal (PoN), and (iv) CH Annotation—A custom annotation script utilizing several external data sources to define CH pathogenicity. The output of the pipeline are three files that separate the variants into (i) those that pass all filters, (ii) variants that are artifacts as defined by the false positive filtering or possible germline variants, and (iii) variants that are recommended for manual review.

ArCH is a variant calling pipeline that consists of four major components: (i) the UMI consensus calling and sequence building, (ii) variant calling and basic filtering, (iii) variant annotation, (iv) and post-pipeline filtering (Fig. 1). The first three modules are encapsulated within the ArCH WDL Pipeline and has an average processing run time of approximately 5 h using the default settings. The final post-pipeline filtering step generates three separate outputs: (i) high confidence variants that pass all associated filters, (ii) variants exhibiting characteristics of artifacts or have low confidence in their annotation support, and (iii) variants that are identified as artifacts. ArCH was developed using the Workflow Development Language (WDL) v1.0 (Voss *et al.* 2017) for integration with Cromwell v69 (Voss *et al.* 2017) which was successfully configured to run on three different environments: (i) IBM’s Spectrum LSF, (ii) Google Cloud Project hosted Cromwell v71 Server, and (iii) TerraBio’s Cloud-Based Platform. As of June 2023, ArCH has been configured and tested to work on Cromwell v86 and WDL v1.1.

### 2.4 Genomic analysis and variant calling

After demultiplexing, FASTQ files were converted into unaligned BAM files. The 8 bp UMI was then removed from either the sequence or the read name (depending on the sequencing technology) and used to tag the unaligned read. BWA-MEM v0.7.15-r1140 was used to align the reads to the hg38 reference genome (Li 2013) and the resulting BAMs were processed using Fulcrum Genomics’ consensus calling workflow using their toolset fgbio v1.3.0–0 (http://fulcrumgenomics.github.io/fgbio/). Aligned reads were grouped as read families by their UMI and reference position. For each read family with a minimum of one read, a single consensus sequence was constructed to represent the entire read family with a per base error rate of 10%. Consensus sequences in which 5% of the bases in the read were ambiguous or had over 50% no-call bases (N), were filtered and removed. Remaining consensus sequences were then realigned using BWA-MEM v0.7.15-r1140 again to improve alignment accuracy by reducing the rate of false alignments from the PCR amplification step introduced during library preparation, which were removed by the consensus sequencing building (Li 2013). Following consensus sequencing building, we observed a mean of 19 664× coverage across all samples. Finally, individual base quality scores were readjusted using GATK v4.1.8.1 Base Quality Score Recalibration tool (McKenna *et al.* 2010).

Variant calling was performed using four separate variant callers: Mutect2 v4.2.0.0 (Cibulskis e*t al.* 2013), VarDictJava v1.8.2 (Lai *et al.* 2016), LoFreq2 v2.1.5 (Wilm *et al.* 2012), and Pindel v0.2.5b8 (Ye *et al.* 2009). We selected mutation callers based on head-to-head comparisons of commonly used tools performed by Sandmann *et al.* (2017). Mutect2 and VarDictJava were chosen for their overall high sensitivity and specificity. LoFreq2 was chosen given its higher performance for detecting low VAF variants under 5% (Sandmann *et al.* 2017) and its lower run time compared to other variant callers also optimized for low VAF events (e.g. FreeBayes). Pindel was chosen for its ability to call insertions/deletions (INDELs) of varying lengths. Mutect2 was run according to the GATK best practices workflow except the max-reads-per-alignment-start parameter was removed to prevent down sampling. VarDictJava was run using the default workflow except to increase complex INDEL calling (-X flag). LoFreq2 was run using the recommended base settings with INDEL recalibration. Pindel was run using default settings. All resultant VCF files were normalized using bcftools v1.13 (Danecek *et al.* 2021).

### 2.5 Variant annotation

VCF files produced by Mutect2, VarDictJava, and LoFreq2 were concatenated together, and the canonical transcripts were annotated using Variant Effect Predictor (VEP) version 104 (McLaren *et al.* 2016). Variants were also annotated using gnomAD v2.1.1 exome dataset (Karczewski *et al.* 2020), and gnomAD v3.1.2 genome dataset (Chen *et al.* 2022), ClinVar (Landrum *et al.* 2020), OncoKB v3.10 (Chakravarty *et al.* 2017), and COSMIC v95 (Tate *et al.* 2019). Finally, variants were annotated with the frequency each variant was detected in two previous large CH publications analyzing healthy individuals (Bick *et al.* 2020b) and solid tumor patients (Bolton *et al.* 2020).

#### 2.5.1 Clonal hematopoiesis post-processing filtering criteria

We performed a series of false positive filtering and artifact removal steps following variant calling based upon previous post-variant calling filtering applied by us and others (Koboldt *et al.* 2012, Bick *et al.* 2020b, Bolton *et al.* 2020). We removed the variants based on the following criteria:

1. Variants with a VAF >2% in two or more PoN samples which replicated the PoN filter in Mutect2.
2. Variants in which the proportion of alleles across all PoN samples supporting the variant were not statistically significantly different compared to the proportion of alleles supporting the variant in a given sample. This was assessed by comparing the alternate and reference allele counts in the individual sample compared to the pooled PoN samples using a Fisher’s exact test. Statistical significance was defined as lower than an adjusted *P*-value for multiple hypothesis testing using a Bonferroni correction using the number of base pairs within the panel capture region.
3. Variants that failed VarScan2’s false positive filter (Koboldt *et al.* 2012).
4. Variants that were specifically called only by VarDictJava that failed Blue Collar Bio’s low VAF and low-quality filters. (https://github.com/bcbio/bcbio_validations/blob/master/somatic-lowfreq/README.md)
5. INDELs >20 bp without support from multiple variant callers, as these are typically misaligned reads based on our prior experience.
6. Variants with evidence from only the forward or reverse strand.
7. Variants with an alternate allele depth <5.
8. Recurrent variants present in >6% of all samples. This cutoff (6%) was set through analysis of prior large-scale CH sequencing studies (Bick *et al.* 2020b, Bolton *et al.* 2020) for the proportion of DNMT3A R882H mutations, which has been previously shown to be the most commonly mutated CH hotspot.
9. Recurrent variants present in >3% of all samples without being previously identified in large-scale CH sequencing studies (Bick *et al.* 2020b, Bolton *et al.* 2020).

#### 2.5.2 Clonal hematopoiesis putative driver classification

We defined CH putative drivers using the following criteria:

1. Truncating mutations within *DNMT3A*, *TET2*, *TP53*, *ASXL1*, and *CHEK2.*
2. Truncating mutations within exon 6 of *PPM1D.*
3. In-frame INDELs within *CHEK2.*
4. Missense mutations present as somatic within COSMIC at least 10 times or at least five times within the “hematopoietic and lymphoid” category.
5. Variants previously reported as CH mutations in studies from healthy or solid tumor patients (Bick *et al.* 2020b, Bolton *et al.* 2020).
6. Variants classified as either oncogenic or likely oncogenic by the OncoKB database.
7. Missense mutations within the same amino acid loci as a previously reported CH variant by Bick *et al.* (2020b) or Bolton *et al.* (2020) with computational evidence of being potentially damaging by SIFT or PolyPhen (Ng and Henikoff 2003, Adzhubei *et al.* 2010).
8. Missense mutations within three amino acid residues or within nine nucleotides of CH hotspot variants from Bick *et al.* (2020b) or Bolton *et al.* (2020). Hotspot mutations were defined as variants that were reported at least five times in either Bick *et al.* (2020b) or Bolton *et al.* (2020); present in COSMIC at least 25 overall, at least 20 times within the “hematopoietic and lymphoid” category, or at least 10 times within the “myeloid” category; and had computational evidence as being potentially damaging by SIFT or PolyPhen.
9. Missense mutations within established hotspot regions for *SRSF2* (amino acid position 95) and *SF3B1* (amino acid positions 622–626, 662–666, 700–704, 740–742).
10. Variants reported as likely pathogenic or pathogenic in ClinVar.

### 2.6 Bona-fide variant identification

#### 2.6.1 Dilution series

Within the AML tumor:normal dilution series, 18 somatic variants in each tumor sample were previously identified as driver mutations using clinical sequencing (Supplementary Table S2). We interrogated these target variants across the subsequent diluted series and observed a decrease in VAF with an increasing normal to tumor ratio. We considered these 18 variants as bona-fide acquired genetic alterations in these tumors. To increase our set of true positive variants, we also queried all tumor samples for somatic and germline genetic variants that were not shared between multiple tumor samples. We then identified tumor:tumor pairings from unique patients showing no somatic overlap and the lowest germline variant similarity. In-silico dilution was then performed on each AML tumor:tumor BAM pairing to create a dilution series at 1:10, 1:100, 1:1000, and 1:5000. We considered 31 total somatic or germline variants as bona-fide alterations for our benchmarking experiments (Supplementary Table S2).

#### 2.6.2 Normal blood validation set

We defined variants with strong support from multiple sequencing approaches as *bona fide* alterations using the following approach. First, we queried the ArcherDX VariantPlex data for variants meeting the following criteria: (i) with a VAF of >0.001, (ii) had an alternative allele depth >5, (iii) passed at least one variant caller, (iv) passed a reduced set of post-variant calling filters including the incomplete read count (IRC) false positive filter (Koboldt *et al.* 2012), and (v) passed the PoN Fisher’s exact test; 715 variants met these criteria. Second, these variants were then interrogated for supporting evidence (>5 alternate reads) in the MyeloSeq™-HD data. A total of 270 variants met both criteria and were considered to be *bona fide* alterations (Supplementary Table S3). A subset of these samples (*N* = 10, with 86 *bona fide* variants) were sequenced using a second orthogonal approach, the Element AVITI™ platform. Out of 86 variants, 85 were also found using this independent sequencing platform, thus confirming our confidence in our ability to select *bona fide* variants in this call set.

#### 2.6.3 Duplex sequencing of technical replicates

Variants that were supported in both technical replicates, which we denote as replicate A and B, and met the following criteria were classified as *bona fide* variants. First, we required that the variant meet the following criteria in replicate A: (i) passed by at least one variant caller, (ii) passed the PoN Fisher’s exact test, (iii) had a VAF of >0.001, and (iv) passed a reduced set of post-variant calling filters including the IRC false positive filter. Second, we required the variant show some level of evidence (>5 alternate reads) in replicate B. A total of 43 variants were detected meeting these criteria and considered to be *bona fide* variants (Supplementary Table S8). We performed CH variant calling using ArCH in replicate B. Variants that passed ArCH but were not classified as *bona fide* variants were considered false positives.

#### 2.6.4 Statistical prediction of artifacts

We used extreme gradient boosting (XGBoost) to develop a predictive model for artifact filtering. This method based on a gradient boosted collection of weighted and connected decision trees (Chen and Guestrin 2016). The primary advantage XGBoost has over other commonly used machine learning methods is that it allows for missing data in its algorithm without the need to provide placeholder values or impute of missing data. Features generated from the three variant callers (Mutect2, VarDictJava and LoFreq2) along with our post-calling false positive filters were used as the inputs to the model; using binary-logistic as the objective function and the default values for all hyperparameters without tuning. We trained models on the normal blood validation set using stratified 10-fold cross validation for classification tasks using scikit-learn model selection package. Truth labels were derived from orthogonal sequencing as previously described. The normal blood sample set was chosen as the training dataset given the larger number of *bona fide* variants. The best performing model from cross validation was saved and used for prediction in the AML tumor dilution dataset.

### 2.7 Panel of normal size optimization

To evaluate the impact of the size of the PoN on ArCH’s ability to differentiate *bona fide* variants from sequencing artifacts, we simulated various PoN sizes, ranging from 1 to 27 samples. We addressed potential sample selection bias by incorporating all possible combinations of normal samples from our original PoN (*n* = 27). For each PoN combination, we conducted a Fisher’s exact test comparing the proportion of alternate counts to reference counts in the PoN compared to the proportion of alternate counts to reference counts for each *bona fide* variants in the AML dilution series. We computed the proportion of these variants that our PoN filter removed, using a Bonferroni corrected *P*-value of 2.1e−6, taking into account the total target size of the ArcherDX targeted panel (23 650 bp).

## 3 Results

### 3.1 Performance of somatic variant callers in AML tumor dilution series

We detected 91 497 single nucleotide variants (SNVs) across 30 AML tumor: dilution samples that passed at least one variant caller. Most SNVs (99.9%) were supported by one variant caller (Supplementary Fig. S1A). VarDictJava called the vast majority of SNVs that had no support from other variant callers (99.8%, *N* = 91 289). The proportion of SNVs supported by multiple variant callers increased by VAF. Less than 0.1% of mutations with VAF <0.4% were supported by multiple variant callers, increasing to 36.0% for VAF 0.4%–1%, 85.0% for 1%–5% and 100% for mutations with a VAF >5%. Overall, 54 699 INDELs were passed by at least one variant callers. Similar to SNVs, most INDELs were passed by only one variant caller (90.6%) (Supplementary Fig. S1B). Pindel accounted for the majority of singleton calls (90.6%) followed by VarDictJava (6.9%). Due to its lengthy runtime and discrepant calls, it was removed as a core INDEL caller. Of the remaining three variant callers, the proportion of INDELs supported by multiple variant callers increased by VAF. 10.0% of INDELs with VAF <0.1% were supported by multiple variant callers, increasing to 18.2% for VAF 0.1%–0.4%, 50.0% for 0.4%–1%, 57.9% for >1%. In summary, for low VAF events (VAF < 1%) there was marked discordance between variant callers.

We next evaluated the performance of individual variant callers in regard to their sensitivity and PPV. PPV was used over specificity due to the inability to define the total number of possible true negative variants. VarDictJava showed good sensitivity for both SNVs and INDELS >0.1% VAF (98.9%) with sensitivity decreasing to 66.0% for variants with VAFs <0.1% (Supplementary Fig. S2A). While the PPV was high for mutations >1% VAF (91.2%), it was lower for mutations <1% VAF (37.3% for 0.4%–1% VAF, 0.4% for 0.1%–0.4% VAF and 0.04% for <0.1% VAF). LoFreq2 performed similarly to VarDictJava but with a lower sensitivity for mutations <0.4%. Mutect2 showed a lower sensitivity across the range of VAFs, particularly for mutations <1% VAF but a higher PPV for mutations <1% VAF. Thus, no single variant caller showed both a high sensitivity and PPV for variants under 1% VAF. Because of the poor PPV and sensitivity of Pindel for lower VAF INDELs and requiring almost five times longer computational time compared to other callers, we excluded it from further consideration.

To address the limited PPV for individual variant callers for low VAF mutations, we considered a consensus calling approach, classifying variants as to whether they were passed by one or more variant callers. When considering variants that passed at least one variant caller, there was perfect sensitivity (100%) but low PPV below 1% VAF (26.8% for 0.4%–1% VAF, and close to <0.1% for variants below 0.4% VAF) (Supplementary Fig. S3). When considering variants that passed at least 2 variant callers, the sensitivity remained high for variants above 1% (96.8%) but dropped for variants between 0.1 and 0.4% VAF (28.6%) and approached 0 for variants <0.1% VAF. The sensitivity for variants that passed 3 or more variant callers was lower across the range of VAF, being below 25.0% for variants under 0.4% VAF. The PPV increased modestly when applying a consensus approach (passing 2 or 3 variant callers compared to 1 caller) for variants above 1% VAF. However, among variants with VAF under 1%, PPV was substantially higher for a consensus approach. Thus, while we were able to improve PPV of lower VAF mutations by using a consensus calling approach, the sensitivity was poor for variants below 1%.

**Figure 2.**
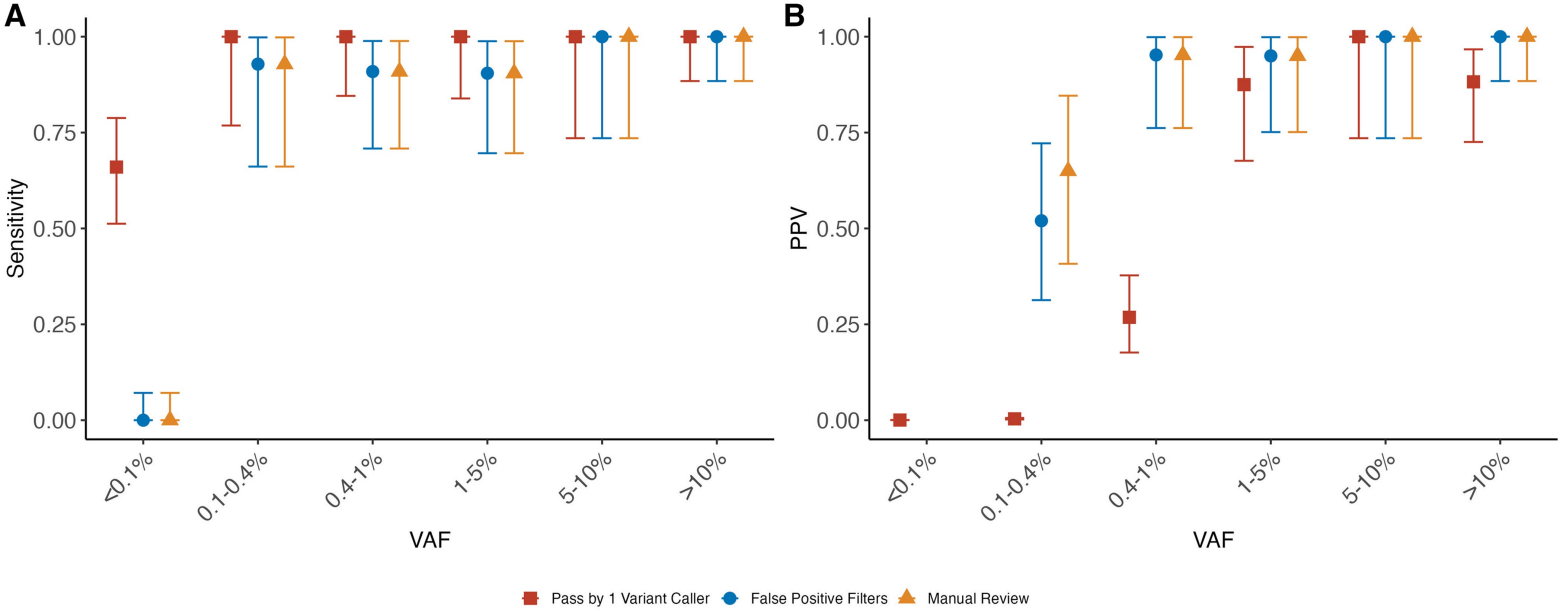
Sensitivity and positive predicted value (PPV) for the AML dilution series. Sensitivity (**A**) and PPV (**B**) for AML dilution series for variants that (i) passed one or more variant callers (square), (ii) passed one or more variant callers and our false positive filters (circle), and (iii) passed one or more variant callers, false positive filters, and was manually reviewed (triangle). Sensitivity was calculated as the number of detected true positives over the total number of *bona fide* mutations within a given VAF category. PPV was calculated as the number of true positives over the number of total positives as defined by the three criteria defined previously within a given VAF category. Error bars show the 95% confidence interval for sensitivity and PPV as obtained by the Clopper–Pearson intervals method.

Given the poor PPV for individual callers and poor sensitivity for a consensus calling approach for variants under 1% VAF, we next applied a series of false positive (FP) filters following variant calling to increase the PPV. Each filter provided an incremental increase in the detection of false positives with the majority of false positives being captured by the panel of normal test (Supplementary Table S4). Following application of these filters, we saw a significant improvement in the PPV for low VAF variants: PPV 95.2% for variants between 0.4%–1% VAF and 65.0% for 0.1%–0.4% VAF (Fig. 2 and Supplementary Table S5). For mutations between 0.1 and 0.4% VAF, PPV increased when a variant was supported by multiple callers (52.0% for passed by 1, 66.7% for passed by 2, and 100% for passed by 3) (Supplementary Fig. S4). For variants above 0.1% VAF, following application of FP filters, the sensitivity remained above 95.0% for variants passed by one or more callers (Fig. 2, Supplementary Fig. S4, and Supplementary Table S5). However, all variants below 0.1% VAF failed our post-variant calling filters driven largely by the PoN test (Supplementary Table S4). The mean VAF of these variants was 0.045% in our PoN and 0.037% in our samples. We considered whether we might rescue *bona fide* variants at VAFs <0.1% through restricting our call set to established hotspot CH/Myeloid neoplasm mutations and relaxing our *P*-value cutoff for the PoN statistical test. However, sensitivity was only marginally improved to 11.0% and PPV remained under 25.0% (Supplementary Fig. S5). In summary, following application of a series of FP filters customized for CH we were able to improve the PPV using a multi-caller approach. However, we were unable to reliably detect mutations under 0.1% VAF using the ArcherDX platform as this approached the level of sequencing error in our PoN.

Since we observed that a large proportion of artifacts were being filtered due to the PoN filter, we examined the effect of the PoN panel size on our ability to distinguish *bona fide* variants from artifacts (Supplementary Fig. S6). Most variants whose read counts were not significantly different than those the PoN were below 0.2% VAF. However, we observed a range of VAFs at which a given variant was filtered by the PoN filter, between 0.02% and 5% VAF reflecting the range of intrinsic sequencing error throughout positions in our panel. Using our analysis of varying PoN sizes, we found that after 8 PoN samples, any additional increase in the PoN size no longer significantly decreased the number of *bona fide* variants failing the PoN filter (Supplementary Fig. S6).

Many somatic mutation calling approaches including ones performed specifically for CH variants include a manual review. This review consists of evaluation by an expert with visual or computational inspection using IGV or other bioinformatic tools for variants that are considered possible artifacts. Among variants that were passed by one or more variant callers, and which passed the additional FP filters, one reviewer (KB) performed manual curation. Out of the 146 variants, 5 variants were considered to be artifacts: 2 were complex INDELs without any additional variant caller support and 3 were mutated in multiple samples but not previously reported in tumor or CH published datasets (Supplementary Table S3). All 5 of these variants flagged as potential artifacts were confirmed to be false positives between 0.1% and 0.4% VAF. Following application of the manual review, we saw a modest improvement in the PPV for mutations between 0.1% and 0.4% VAF from 52.0% to 65.0% (Fig. 2 and Supplementary Table S6).

We further explored the remaining seven false positive mutations that passed our variant calling, post-calling FP filters and manual review. All of these were present at low VAF in the tumor sample (<0.5%). Because these were not detected in any AML dilution samples, we considered them to be an artifact based on our definition of *bona fide* variants. However, given the low level of alternative read support in the tumor, it is possible that some of these variants could represent *bona fide* low-level CH events in the tumor that were not detected in AML dilution series and thereby misclassified as artifacts.

### 3.2 Performance of ArCH for CH detection in normal blood samples

**Figure 3.**
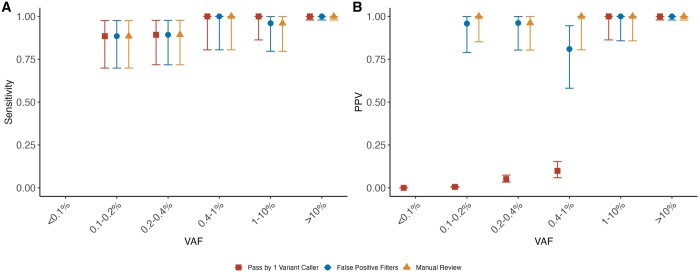
Sensitivity and positive predicted value (PPV) for the normal blood samples. Sensitivity (**A**) and PPV (**B**) for CH detected in normal blood samples for variants that (i) passed one or more variant callers (square), (ii) passed one or more variant callers and our false positive filters (circle), (iii) passed one or more variant callers, false positive filters, and was manually reviewed (triangle). Sensitivity was calculated as the number of detected true positives over the total number of *bona fide* mutations within a given VAF category. PPV was calculated as the number of true positives over the number of total positives as defined by the three criteria defined previously within a given VAF category. Error bars show the 95% confidence interval for sensitivity and PPV and PPV as obtained by the Clopper–Pearson intervals method.

In the 31 orthogonally sequenced blood samples from individuals without hematologic malignancy, we identified 382 345 variants (SNP and INDELs) that were passed between Mutect2, VarDictJava, and LoFreq2. Among these, 269 variants passed our post-variant calling filtering. As seen previously in our AML dilution series, after application of the false positive filters, the PPV for low VAF variants was high (100% PPV for variants above 1% VAF and 91.0% PPV between 1% and 0.2% VAF) while retaining 98.0% sensitivity for variants above 1% VAF and 92.6% between 1% and 0.2% VAF. Manual review slightly improved the PPV for low VAF variants (98.7% for variants below 1% VAF) (Fig. 3B). As in the AML dilution samples, the variants flagged as potential false positives on manual review were complex INDELs with support from one variant caller (*N* = 3).

**Figure 4.**
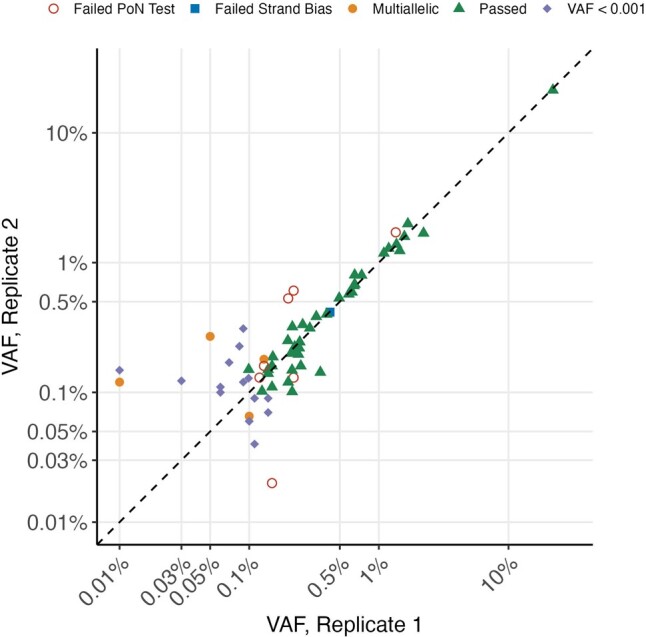
Replication accuracy for technical replicates using ArCH. Replication of *bona fide* CH variants in normal blood samples. True *bona fide* CH mutations that passed ArCH in both technical replication samples are shown as triangles. All other *bona fide* CH mutations that passed in only one of the two replication samples are labeled according to the reason for failing.

Finally, in order to validate the reproducibility of ArCH as applied to the ArcherDX VariantPlex panel, we performed the same variant calling selection and filtering methodology on the 21 normal blood samples with technical duplicates. Among 67 CH variants that were passed by ArCH in one or more replicates, 40 variants were passed in both. In total, 27 variants were not replicated. The majority (85.2%) of these variants were present below 0.2% VAF. The most common reasons for a variant failing in the corresponding replicate was due to the PoN filter (*N* = 4), the 0.1% VAF cut-off threshold (*N* = 15), or having another alternate allele called (*N* = 4) (Fig. 4). For the majority of cases, CH variants did not replicate due to the large impact of small changes in alternative allele counts on VAF for low VAF mutations. Correspondingly we saw a decrease in the correlation between VAF for variants under 0.2% VAF between technical replicates (correlation coefficient = 0.036) compared to variants above 0.2% VAF (correlation coefficient = 0.99). Among the four variants above 0.2% VAF that did not replicate, three failed the PoN filter and were in low complexity regions (one, the ASXL1 G646Wfs*12 mutation and two PPM1D deletions). In summary, ArCH showed excellent reproducibility and precision for measurement of CH using ArcherDX VariantPlex above 0.2% VAF.

### 3.3 Performance of ArCH for CH detection using duplex sequencing

**Figure 5.**
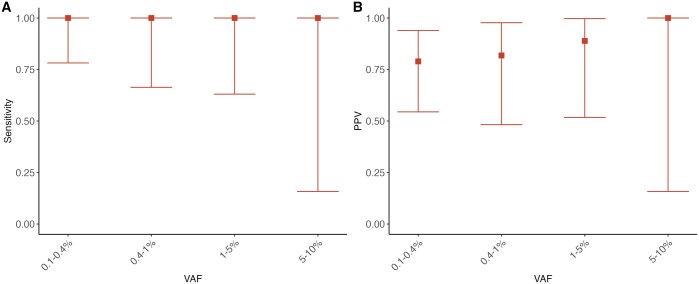
Sensitivity and positive predicted value (PPV) for the replication samples. Sensitivity (A) and PPV (B) for CH variants using ArCH in blood samples using duplex targeted sequencing of a 31 gene panel. Sensitivity was calculated as the number of variants passed by ArCH divided by the total number of *bona fide* mutations within a given VAF category. PPV was calculated as the number of true positives over the number of *bona fide* mutations divided by the total number of variants passed by ArCH. Error bars show the 95% confidence interval for sensitivity and PPV and PPV as obtained by the Clopper–Pearson intervals method.

Among five samples, we identified a total of 192 variants (SNP and INDELs) that passed at least one variant caller. Out of these, 57 passed post-variant calling filtering. 16 of these variants were detected at VAFs <0.1%. ArCH showed excellent PPV (80.4% for <1% VAF and 94.5% for >1% VAF) while maintaining 100% sensitivity (Fig. 5). For two samples, a third technical replicate was available, which we denote as replicate C. We queried the “false positive” variants (i.e. variants that were present in replicate B but not replicate A) for evidence of support in the third replicate, replicate C. Among the 8 possible “false positive” variants below 1% VAF, 5 were supported by at least five reads in replicate C. Thus, in some cases, these “false positive” variants that were passed by ArCH may actually represent true positives.

### 3.4 Machine learning

We sought to characterize whether a machine learning model incorporating variant caller flags, FP filters and variant specific features might improve the balance of sensitivity and PPV. We used XGBoost to develop a prediction model in the healthy blood cohort with training in the AML tumor: normal dilution series. In the validation set, this model showed strong discrimination between true and false positive variants (AUC of 0.99) (Supplementary Fig. S7). The most important features for this model included the PoN filter, the frequency of the variant in previously published CH datasets, and the variant position within the read (Supplementary Table S7). We compared the sensitivity and PPV for various cutoffs of our artifact score generated from the XGBoost model (Supplementary Fig. S8). While the model showed excellent sensitivity and PPV above 1% for a variety of cutoffs, the performance for variants below 1% decreased. For variants below 1% VAF, no single cutoff showed an improved balance of sensitivity or PPV over multiple variant calling, FP filters, and a limited manual review. Thus, the XGBoost model did not result in improved performance above application of our FP filters and a limited manual review.

## 4 Discussion

We developed ArCH, an end-to-end pipeline for the analysis of UMI-based sequencing platforms tailored for CH calling and interpretation which integrates unaligned read quality preprocessing, consensus sequence building, alignment, variant calling, custom filtering and annotation (Fig. 1). Through biological data, we demonstrate that ArCH outperforms traditional single variant callers at detecting and differentiating *bona fide* CH mutations from artifacts, particularly for CH mutations below 1% VAF. In order to further facilitate its use in clinical and translational research settings, we have integrated a variant annotation workflow which leverages pre-existing variant annotation tools such as VEP in conjunction with external curated cancer databases and published CH datasets. These additional annotations are further leveraged during the post-pipeline filtering step for a more accurate identification of CH driver variants.

ArCH is optimized for flexibility allowing multiple different NGS input types and customizable parameter tuning to fit different research questions or datasets. In order to simplify variant interpretation, we have designed ArCH to output three non-overlapping sets of variants; high confidence variants that passed all filtering criteria, variants with characteristics similar to artifacts that are recommended for manual review, and variants that failed various filtering criteria. ArCH is currently implemented as a workflow description language (WDL) workflow for portability and reproducibility using pre-installed variant callers that are optimized for efficient execution in parallel to reduce the overall runtime and costs. ArCH is publicly available in Dockstore and portable within a variety of cloud-based workflow managers including TerraBio and DNANexus as well as local high-performance computing clusters.

While we used several sequencing approaches in our validation studies, our benchmarking of ArCH was mainly based upon our nine-gene panel using the ArcherDX’s VariantPlex platform. Differences in sample preparation, gene set, and sequencing approach will lead to variations in artifact profiles as seen in our replication set using a different sequencing technology. However, one of the strengths of our approach is the use of a study-specific PoN which, through providing an empiric estimation of base-specific error rate, can accommodate differences in error profiles. While we anticipate that ArCH should have comparable performance with other UMI-based targeted panels, platform-specific benchmarking would further facilitate the generalizability of ArCH. This comparison would be particularly important for emerging technologies, including single cell sequencing and long-range sequencing that have different artifact profiles. While the framework of ArCH would be applicable for other platforms, modifications of the variant calling and filtering strategy would be required. Several of our filters including for example the removal of, long, complex Indels with limited support, may be overly conservative, removing in some cases *bona fide* variants. With the continued development of long-read sequencing, the accurate detection of complex structural events driving CH and other forms of mosaicism will be feasible. Further refinement of variant calling and filtering strategies including validation using benchmarking experiments will be needed.

In summary, we present ArCH, a comprehensive pipeline for the analysis of UMI-based CH sequencing studies. We anticipate that ArCH will enhance the rigor and reproducibility of CH calling, in particular studies aimed at detecting low VAF events.

## Supplementary Material

btae121_Supplementary_Data

## Data Availability

The sequencing data for the samples drawn from the Health Professionals Follow-up Study are available through a project proposal as described at https://www.hsph.harvard.edu/hpfs/for-collaborators/. All other original data used in this study will be shared on reasonable request to the corresponding author. All code and data used to generate the results presented in this manuscript is publicly available on GitHub at https://github.com/kbolton-lab/ArCH/.
