# Supplementary material for: ArCH: improving the performance of clonal hematopoiesis variant calling and interpretation
Source: Bioinformatics. 2024 Mar 14;40(4):btae121. doi: 10.1093/bioinformatics/btae121 (PMC11014783; doi:10.1093/bioinformatics/btae121)
Supplement: btae121_Supplementary_Data [file btae121_supplementary_data.zip › SupplementaryFigures_03152024.pdf]

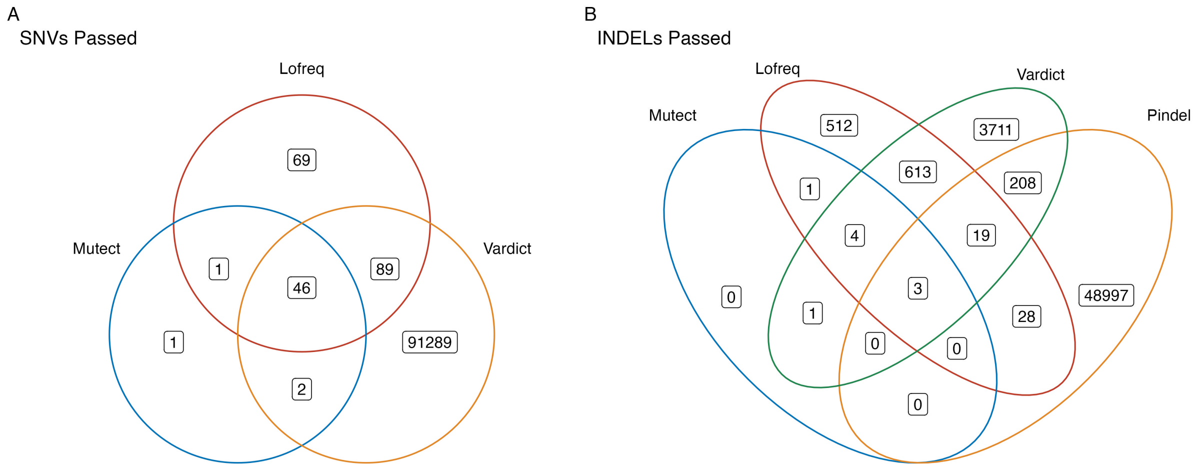

Supplementary Figure 1. Total number of variants passed by each variant caller within the AML dilution series data separated into A) SNVs and B) INDELs. PINDEL is an INDEL specific variant caller that does not report SNVs.

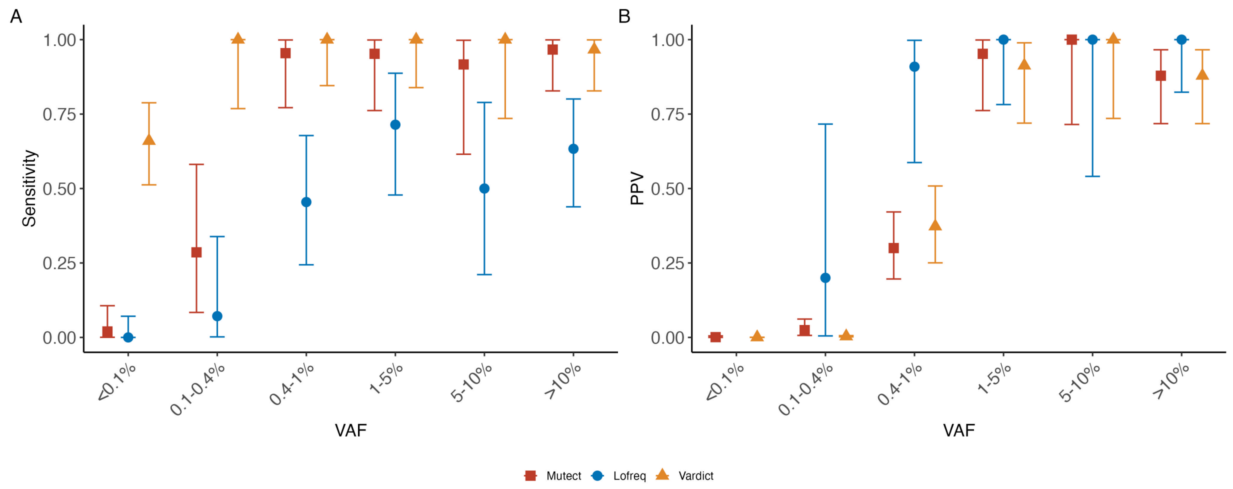

Supplementary Figure 2. Sensitivity and positive predictive value (PPV) of Mutect2 (square), LoFreq2 (circle), and VarDict (triangle) in the AML dilution series for variants. Sensitivity was calculated as the number of detected true positives over the total number of *bona fide* mutations within a given variant allele fraction (VAF) category. PPV was calculated as the number of true positives over the number of total passed variants as defined the individual variant caller's internal filters within a given VAF category. Error bars show the 95% confidence interval for sensitivity and PPV as obtained by the Clopper-Pearson interval method.

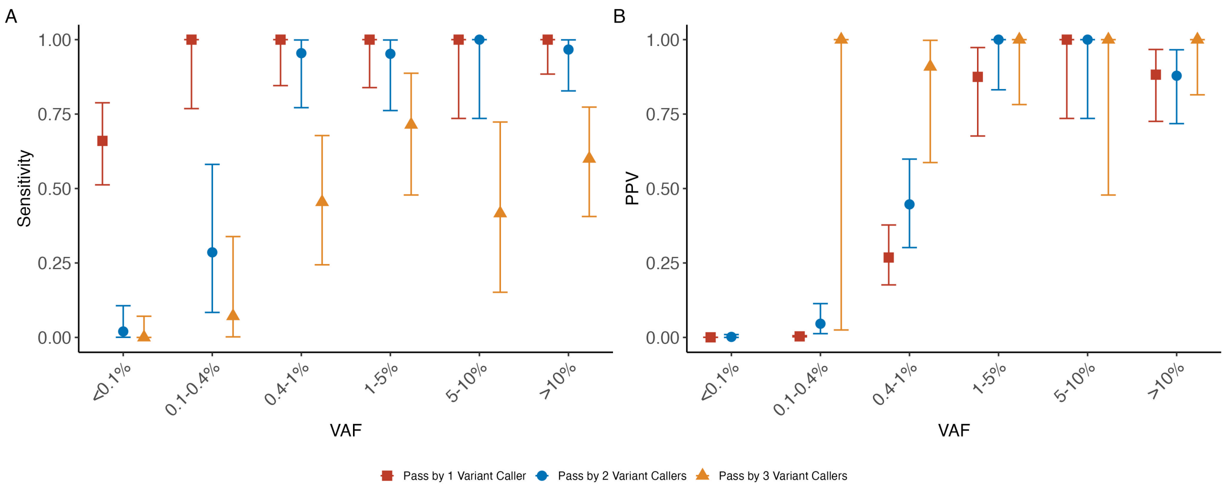

Supplementary Figure 3. Sensitivity and positive predictive value (PPV) for CH detected in the AML dilution series for variants that were 1) passed by a single variant caller (square), 2) passed by two or less variant callers (circle), and 3) passed by all three variant callers (triangle). Sensitivity was calculated as the number of detected true positives over the total number of *bona fide* mutations within a given variant allele fraction (VAF) category. PPV was calculated as the number of true positives over the number of total positives as defined by the three criteria defined previously within a given VAF category. Error bars show the 95% confidence interval for sensitivity and PPV as obtained by the Clopper-Pearson interval method.

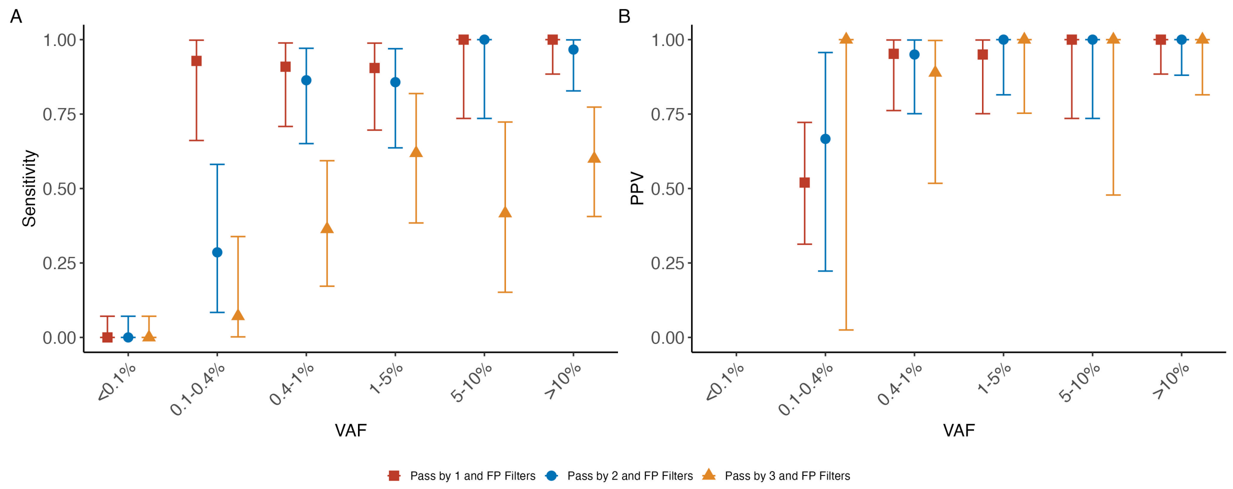

Supplementary Figure 4. Sensitivity and positive predictive value (PPV) for CH detected in the AML dilution series for variants that were 1) passed by a single variant caller that had additionally passed the false positive filters (square), 2) passed by two or less variant callers that had additionally passed the false positive filters (circle), and 3) passed by all three variant callers while additionally passing the false positive filters (triangle). Sensitivity was calculated as the number of detected true positives over the total number of *bona fide* mutations within a given variant allele fraction (VAF) category. PPV was calculated as the number of true positives over the number of total positives as defined by the three criteria defined previously within a given VAF category. Error bars show the 95% confidence interval for sensitivity and PPV as obtained by the Clopper-Pearson interval method.

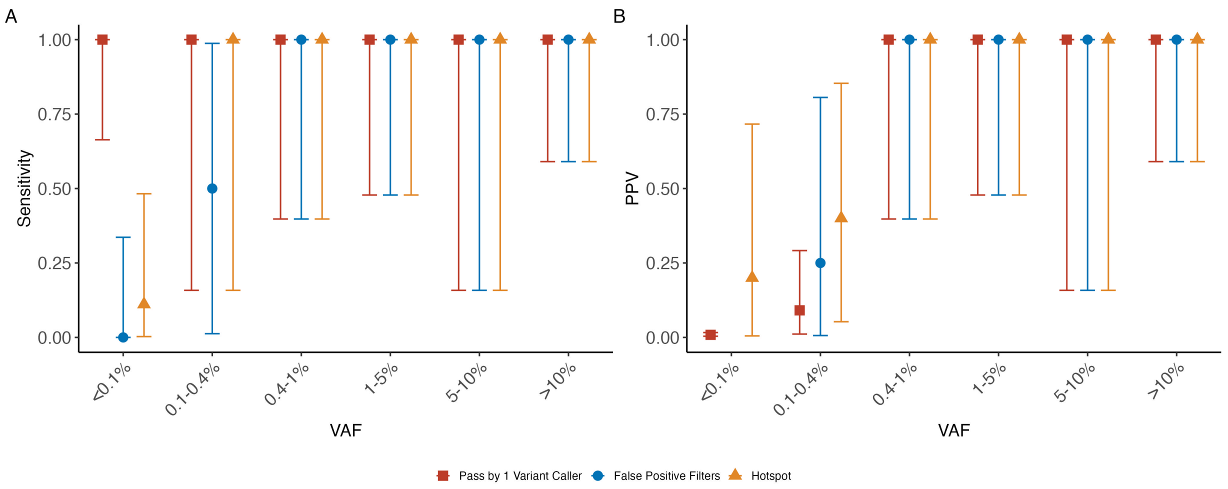

Supplementary Figure 5. Sensitivity and positive predictive value (PPV) for CH hotspots detected in the AML dilution series for variants that were 1) passed one or more variant callers (square), 2) passed one or more variant callers while additionally passing the false positive filters (circle), and 3) passed one or more variant callers and the additional false positive filters but having the PoN false positive filter's Bonferroni corrected p-value cut-off being significantly reduced for specific hotspot loci (triangle). Sensitivity was calculated as the number of detected true positives over the total number of *bona fide* mutations within a given variant allele fraction (VAF) category. PPV was calculated as the number of true positives over the number of total positives as defined by the three criteria defined previously within a given VAF category. Error bars show the 95% confidence interval for sensitivity and PPV as obtained by the Clopper-Pearson interval method. CH hotspots were defined as variants that were reported at least 5 times in either Bick, Weinstock, *et al.* 2020 or Bolton *et al.* 2020 and subsequently reported in COSMIC at least 25 times or at least 20 times within the “hematopoietic and lymphoid” category or at least 10 times within the “myeloid” category.

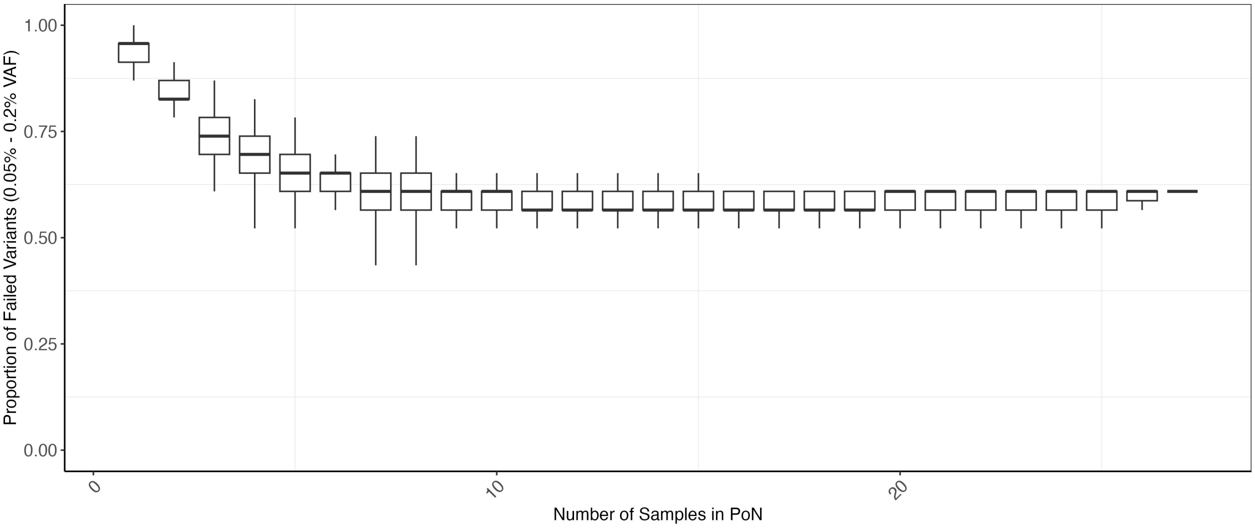

Supplementary Figure 6. The median proportion of *bona fide* variants in the AML dilution series that failed the Fisher's exact test using a Bonferroni corrected p-value of $2.1e^{-6}$ based upon the panel of normal (PoN) sample size. Boxplot lines represent the 1st quartile, the median and the 3rd quartile.

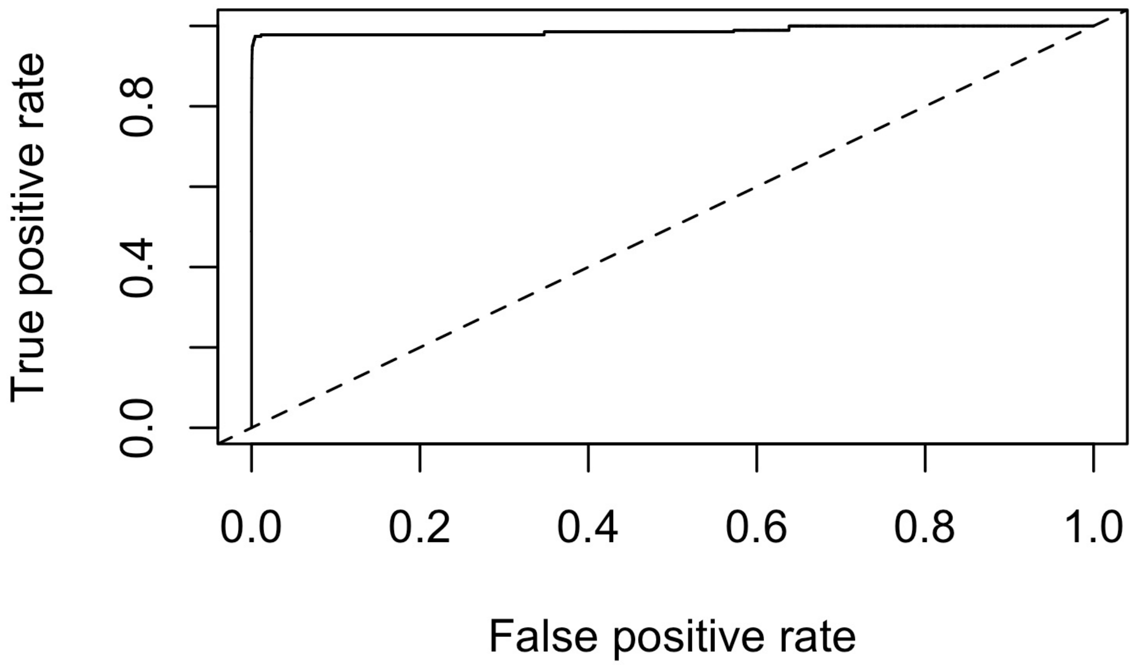

Supplementary Figure 7. Receiver operator curve for discriminating 270 confirmed *bona fide* variants from 382,368 total variants using the XGBoost prediction model in the normal blood samples set.

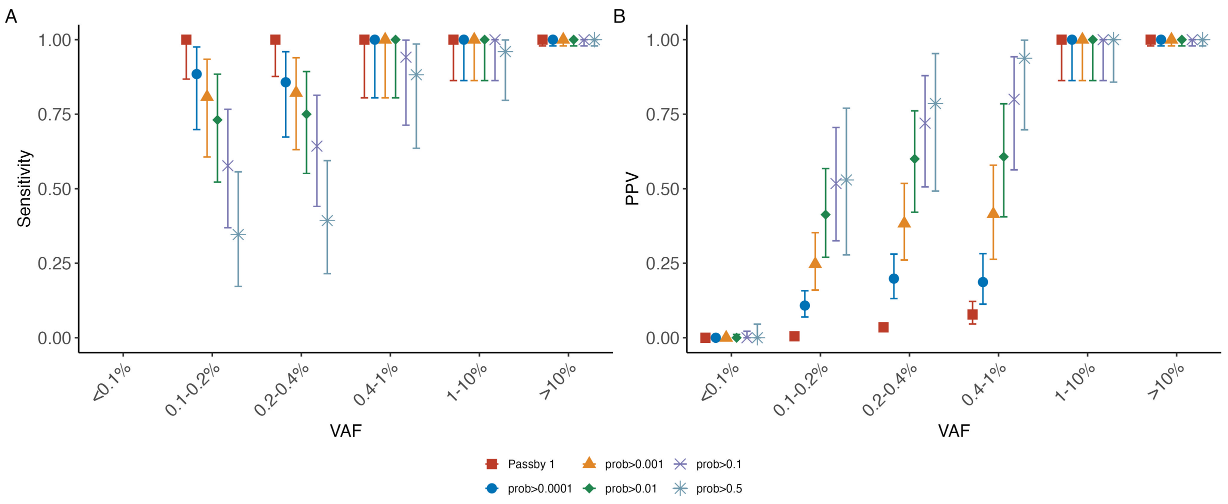

Supplementary Figure 8. Sensitivity and positive predictive value (PPV) for CH detected in normal blood samples for variants that was 1) passed one or more variant callers (square), 2) had a predicted probability score of greater than 0.0001 (circle), 3) had a predicted probability score greater than 0.001 (triangle), 4) had a predicted probability score greater than 0.01 (diamond), 5) had a predicted probability score greater than 0.1 (cross), and 6) had a predicted probability score greater than 0.5 (star). Sensitivity was calculated as the number of detected true positives over the total number of *bona fide* mutations within a given variant allele fraction (VAF) category. PPV was calculated as the number of true positives over the number of total positives as defined by the six criteria defined previously within a given VAF category. Error bars show the 95% confidence interval for sensitivity and PPV as obtained by the Clopper-Pearson intervals method.
